# Experimental Warming Does Not Change Fluctuating Asymmetry in Three Willow Species

**DOI:** 10.1002/ece3.72574

**Published:** 2025-11-30

**Authors:** Dmitry E. Gavrikov, Vitali Zverev, Mikhail V. Kozlov

**Affiliations:** ^1^ Pedagogical Institute Irkutsk State University Irkutsk Russia; ^2^ Department of Biology University of Turku Turku Finland

**Keywords:** fluctuating asymmetry, leaf size, open‐top chambers, *Salix caprea*, *Salix myrsinifolia*, *Salix phylicifolia*, specific leaf area

## Abstract

Fluctuating asymmetry (FA) is often proposed as an early warning indicator of subtle changes in plant functioning. Here, we tested whether leaf FA responds consistently to the alleviation of cold stress in three boreal willow species— *Salix caprea* , *S. myrsinifolia* and *S. phylicifolia*. We enclosed 10 naturally growing individuals of each species in open‐top chambers at budburst and compared their leaf traits to those of unenclosed control plants after leaf development had ceased. All measurements were conducted blind to treatment. Willows in open‐top chambers showed a 9% increase in specific leaf area, indicating that the 1°C–2°C warming within chambers affected leaf development. However, neither leaf length nor FA responded significantly to the warming treatment. FA also did not differ among species or individual plants, suggesting that it may reflect statistical noise rather than a reliable biological signal in this context. These findings add to growing concerns that many reported FA responses to environmental change may result from confirmation bias—an issue that can be mitigated by adopting blind measurement protocols.

## Graphical abstract

We tested whether leaf fluctuating asymmetry (FA) responds to experimental warming in three boreal willow species using open‐top chambers and blind measurements. While warming increased specific leaf area, neither leaf length nor FA showed significant responses, and FA did not differ among species or individuals. These results suggest that FA may represent statistical noise rather than a reliable indicator of environmental stress.

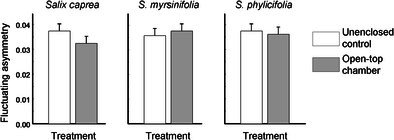

## 1 Introduction

Over the past century, global surface temperatures have risen markedly, with particularly rapid and pronounced warming occurring in high‐latitude and alpine environments (IPCC 2021). Climate projections consistently indicate that disproportionate warming will continue in these usually cold‐limited regions in the coming decades (Post et al. 2019), highlighting a need to anticipate its biological consequences.

In cold‐limited ecosystems, plant growth is typically constrained by short growing seasons and low thermal sums; consequently, in these ecosystems, even modest warming can enhance plant growth rate, reproduction and recruitment (Arft et al. 1999; Hollister et al. 2005; Hudson et al. 2011). This enhancement suggests that many cold‐adapted plants, particularly Arctic willows (*Salix* spp.), are currently growing below their climatic potential and that rising temperatures could relieve developmental stress in these plants (Jones et al. 1997; Körner 2016; Buchwal et al. 2019).

Traditional indicators of plant stress, such as slow growth rate, low biomass and poor reproductive success (Dässler 1976; Schubert 1985; Dobbertin 2005), often respond to environmental changes only after critical and sometimes irreversible thresholds are crossed (Chaerle and Van der Straeten 2000; Moustaka and Moustakas 2023). These response lags have increased research interest in early‐warning indicators that could enable detection of subtle physiological or developmental changes in plants before declines in performance are evident (Bussotti and Pollastrini 2021; De Marco et al. 2022). The identification of these early signals could provide policymakers and land managers with additional time to develop mitigation or adaptation strategies.

One feature that has long been promoted as an early indicator of environmental and genomic stress (Zakharov 1990; Parsons 1992; Clarke 1995; Hume 2001; Zakharov and Trofimov 2022; Shadrina et al. 2023) is fluctuating asymmetry (FA)—small, random deviations from perfect bilateral symmetry in morphological traits (Møller and Swaddle 1997; Polak 2003). Observational studies of plants growing in subarctic regions suggest that FA increases during cold growth seasons (Valkama and Kozlov 2001; Kozlov and Zverev 2018) and at high latitudes and elevations (Zverev and Kozlov 2020; Shadrina et al. 2023). Based on these observations, we have hypothesised that moderate warming during leaf development will alleviate cold stress in plants, leading to a consistent reduction in leaf FA across species growing near the treeline. We have also proposed—on theoretical grounds—that leaf FA may be more responsive than leaf size or specific leaf area to temperature changes.

These hypotheses, grounded in decades of observational work, have recently become controversial because the reliability of FA as an indicator has been questioned (Májeková et al. 2024; Kozlov 2025), particularly due to signs of confirmation bias discovered in several supporting studies (Kozlov and Zverev 2018; Kozlov 2024). Here, we propose a rigorously designed, blinded experiment conducted on willows to evaluate these hypotheses and address ongoing debates regarding the utility of FA as an indicator of developmental stress in plants.

## 2 Materials and Methods

### 2.1 Study Objects

The three willow species used in this study ( *Salix caprea* L., *S. myrsinifolia* Salisbury and *S. phylicifolia* L.) differ markedly in their morphological and biochemical features as well as in their distribution, ecological preferences and biotic interactions. Goat willow ( *S. caprea* ) has large, highly pubescent leaves. This relatively shade‐tolerant pioneer species is often found in forest margins, moist woodlands and disturbed sites. The upland species, dark‐leaved willow ( *S. myrsinifolia* ), which has medium‐sized, slightly pubescent leaves, favours cold, mesic to moist habitats, including boreal wetlands and nutrient‐poor peatlands. In contrast, the tea‐leaved willow (*S. phylicifolia*) has small, glabrous leaves and is typically found in moist‐to‐wet, nutrient‐rich environments, such as riverbanks, stream edges and fens. *S. phylicifolia* is more light‐demanding than *S. caprea* , often forming dense thickets in open areas with high soil moisture. All three are Eurasian species, but *S. caprea* spans the broadest temperate–boreal range deep into central Asia, whereas *S. myrsinifolia* and *S. phylicifolia* are more northern/boreal, with *S. phylicifolia* especially centred on North Atlantic–Fennoscandian regions.

*Salix caprea and S. myrsinifolia* have been intensively studied in terms of relationships between environmental stress, leaf FA and various plant traits, whereas no data appear to be available for *S. phylicifolia*. However, the reported FA patterns in willows are generally not significant, and when significant correlations are found, they tend to be inconsistent (Zvereva et al. 1997; Zvereva and Kozlov 2001; Kozlov et al. 2019).

### 2.2 Experimental Design

This study was conducted at the Central Manor site of the Lapland Biosphere Reserve (67°39′09″ N, 32°38′35″ E; 140 m a.s.l.), located approximately 130 m below and 1 km from the alpine treeline. In this region, average temperatures reach −12.4°C in January and 14.0°C in July, while total annual precipitation is approximately 740 mm. By late winter, snow accumulates to 70–120 cm in spruce forests. The frost‐free period spans 50–100 days, and the cool summer season lasts roughly 2.5 months.

**FIGURE 1:**
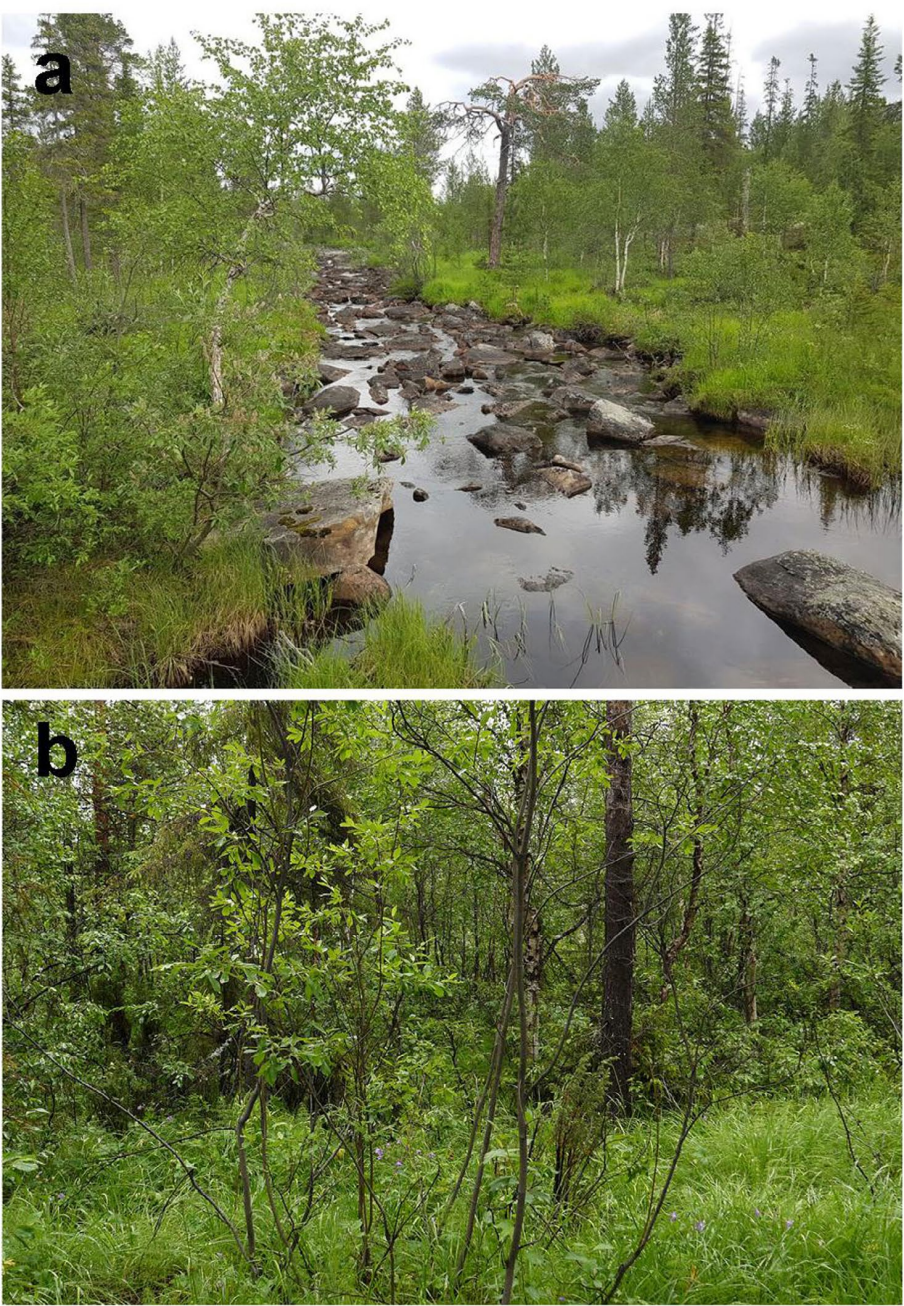
Typical forest near the study site (a; photograph from the archives of the Lapland Biosphere Reserve) and one of the study species, *Salix myrsinifolia* (b; photograph by I. Blinova).

The experimental plot (50 × 50 m size) was originally cleared in the 1970s for use as a helicopter landing pad within a sparse spruce forest (Figure 1). After maintenance ceased in the late 1980s, the area was naturally recolonised by willows.

We selected 20 individuals per willow species, each between 50 and 120 cm tall, and randomly assigned them to either ambient conditions or the open‐top chamber (OTC) treatment. The OTCs were rectangular structures (80 × 80 cm at the base and 160 cm tall) constructed from 120 μm polyethylene film mounted on metal frames. They were installed on 2 June 2000, shortly after budburst in willows. Prior assessments (BASIS 1999) indicated that these chambers consistently raised air temperature by approximately 1°C–2°C compared to ambient conditions.

On 8 July 2000, once the willow leaves had reached their final size, we haphazardly collected 10 leaves from each individual, prioritising leaves located midway along the current year's shoots. The collector was blinded to the study hypothesis. The leaves were press‐dried and mounted as standard herbarium specimens on sturdy A4 paper (210 × 297 mm) in preparation for scanning (Data S1 in Data Availability section).

### 2.3 Leaf Measurements

All measurements were performed using the freeware ImageJ 1.54 g program (NIH), available at http://imagej.org. Image files were anonymised to ensure that the measurer (D.E.G.) was blinded to the treatment applied to each sample. Based on the image dimensions (2480 × 3507 px), the measurement tool in ImageJ was calibrated using the ‘Set Scale’ function. Measurements were taken using the ‘Line’ tool.

Leaf blade length was measured as the distance from the tip to the base of the blade, excluding the petiole. A line perpendicular to the main vein was drawn from the midpoint of the line connecting these landmarks using an ImageJ macro. The distances from the main vein to the left and right edges of the blade were then measured along this line to the nearest 0.01 mm. All values were recorded in the Results Manager.

Each measurement was conducted twice, with a 1‐week interval. The second set of measurements was performed blindly with respect to the first set (Data S2 in Data Availability section).

After scanning, every second leaf was removed from the sheet. The samples of five leaves per plant were dried at 105°C for 24 h and weighed to the nearest 0.1 mg (Data S3 in Data Availability section). The specific leaf area (SLA) was calculated as the ratio of leaf area to dry mass.

### 2.4 Data Analysis

We evaluated the data validity and reproducibility by conducting separate validation analyses for each willow species. Adhering to the guidelines outlined by Palmer and Strobeck (2003), we implemented a mixed‐model ANOVA, treating the leaf side (right or left) as a fixed factor and the individual plant as a random factor. This allowed us to assess the presence of FA and directional asymmetry (DA) in leaf shape. In species in which the mixed‐model ANOVA indicated a significant difference between the right and left sides—evidence of DA—we compared the DA value to the FA4a index, calculated as follows:

(1) $$\mathrm{FA}4a=0.798\sqrt{\mathrm{var}\;\left(R-L\right)}$$

where *R* and *L* refer to the right and left half‐sides of leaf lamina (Palmer and Strobeck 2003). Measurement reproducibility was quantified using the ME5 index, as follows:

(2) $$\mathrm{ME}5=\left(\mathrm{MSi}-\mathrm{MSm}\right)/\left(\mathrm{MSi}+\mathrm{MSm}\right)$$

where MSi represents the mean square of the side × individual interaction, and MSm corresponds to the measurement error mean square derived from two repeated measurements per side per leaf (Palmer and Strobeck 2003). This metric captures the proportion of side variation attributable to FA relative to total side variation (inclusive of measurement error). We also computed ME1, the mean absolute difference between repeated measurements on the same leaf side, calculated as follows:

(3) $$\mathrm{ME}1=\sum \left|M1-M2\right|/n$$

where M1 and M2 are the two independent values and *n* is the number of paired observations (Palmer and Strobeck 2003).

FA was computed using the following formula:

(4) $$\mathrm{FA}=2\times \left|\mathrm{WL}-\mathrm{WR}\right|/\left(\mathrm{WL}+\mathrm{WR}\right)$$

where WL and WR represent the measurements of the left and right halves of the same leaf. This size‐adjusted metric was employed due to the finding of a significant positive correlation between the absolute left–right difference and leaf width in *S. myrsinifolia* (*r* = 0.25, *n* = 198, *p* = 0.0003) and *S. phylicifolia* (*r* = 0.29, *n* = 198, *p* < 0.0001), although no similar correlation was detected in *S. caprea* (*r* = 0.08, *n* = 200, *p* = 0.29). For each leaf, the two independently derived FA values were averaged to yield a single estimate. Only after this step were the anonymised leaf measurements linked to the corresponding experimental treatments.

The sources of variation in FA, leaf length and SLA were analysed using mixed‐model ANOVA (SAS GLIMMIX procedure, type III sum of squares). In the models, treatment (OTC vs. unenclosed control) and willow species were included as fixed effects, while individual plants nested within species were treated as a random effect. Since SLA was measured at the plant level, the random effect was not included in the SLA analysis. Pairwise treatment differences were assessed using Duncan's multiple range test (*α* = 0.05; SAS Institute 2009). Standard errors and denominator degrees of freedom were adjusted according to the method described by Kenward and Roger (2009).

## 3 Results

### 3.1 Data Validation

A significant interaction between leaf side and plant individual was detected in all three willow species (Table 1), confirming both the presence of FA in leaf width and the adequacy of measurement precision to detect it. *Salix myrsinifolia* and *S. phylicifolia*, but not *S. caprea* , also exhibited DA (Table 1). However, the magnitude of DA was substantially smaller than the corresponding FA4a index, indicating that the DA contribution to the overall variation in leaf width was minimal and could reasonably be disregarded.

**TABLE 1:** Basic statistics on repeated blind measurements of the left and right leaf halves of study plants (SAS GLIMMIX procedure, type III sum of squares).

Species	DA^a^, mm	FA4a	Source of variation						Reproducibility	
			Side			Side × Individual			ME1, mm	ME5
			df	*F*	*p*	df	*F*	*p*		
*Salix caprea*	−0.14	2.28	1, 199	1.46	0.23	199, 400	92.1	< 0.0001	0.14	0.979
*Salix myrsinifolia*	−0.14	1.50	1, 197	4.33	0.04	197, 396	114.2	< 0.0001	0.09	0.982
*Salix phylicifolia*	−0.18	0.96	1, 197	16.9	< 0.0001	197, 396	38.2	< 0.0001	0.10	0.947

^a^ DA, average directional asymmetry.

### 3.2 Treatment Effects

**FIGURE 2:**
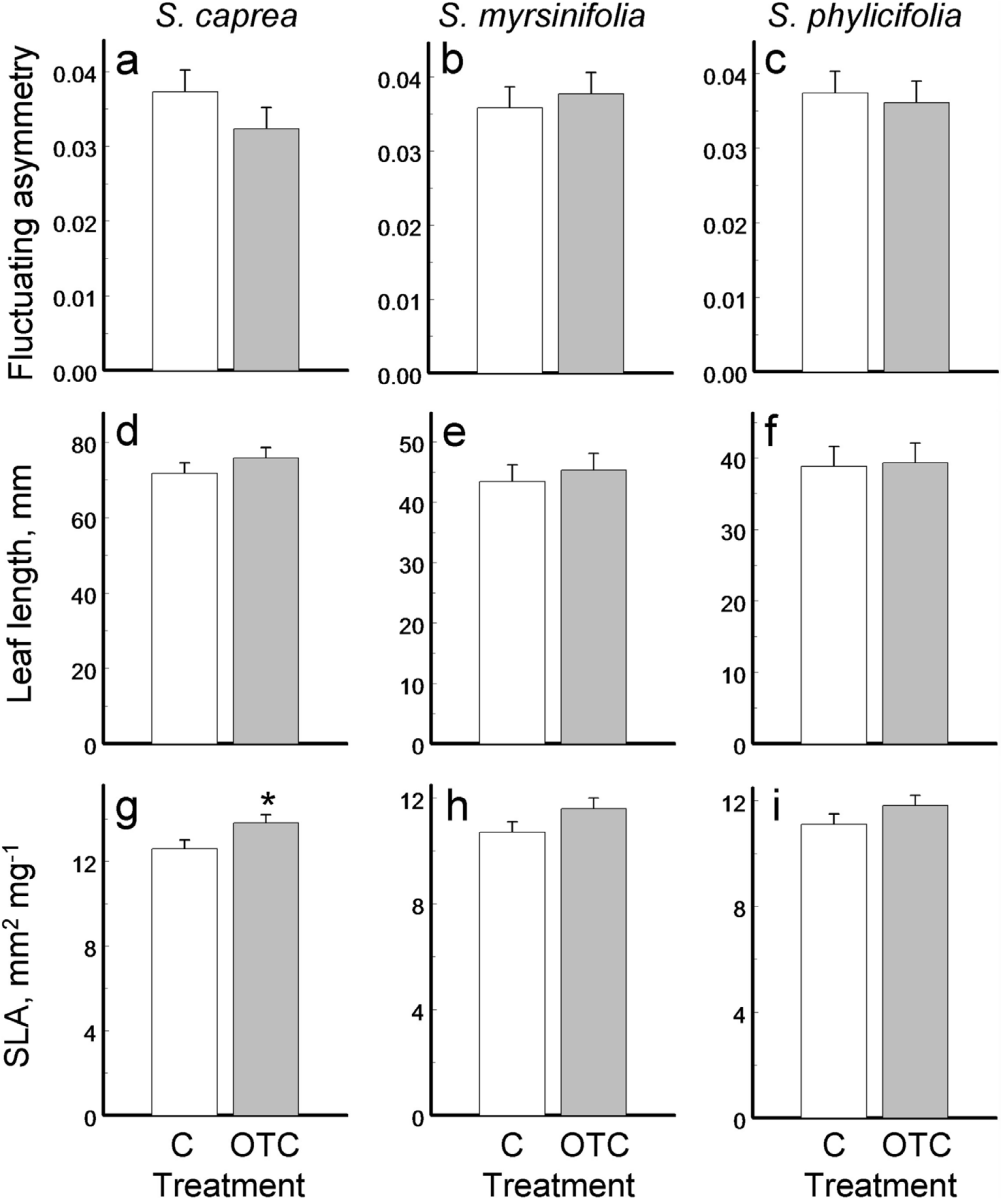
Mean ± SE (*n* = 10 for each bar) values of leaf fluctuating asymmetry (a–c), leaf length (d–f) and specific leaf area (SLA; g–i) in unenclosed controls (Treatment: C) and in open‐top chambers (Treatment: OTC) of three willow species (a, d, g: *Salix caprea* ; b, e, h: *S. myrsinifolia* ; c, f, i: *S. phylicifolia*). An asterisk indicates a significant (*p* < 0.05) difference between treatments.

The effects of OTC treatment on leaf FA were not statistically significant (Table 2), and their directions varied among the three willow species (Figure 2a–c). Leaf length tended to increase under OTC treatment across all species (Figure 2d–f), although this trend did not reach statistical significance. In contrast, SLA showed a statistically significant increase of 9% in plants enclosed in OTCs (Table 2), although within‐species comparisons revealed a significant effect only in *S. caprea* (Figure 2g–i).

**TABLE 2:** Effects of experimental temperature elevation on fluctuating asymmetry, leaf length and specific leaf area in three willow species (SAS GLIMMIX procedure, type III sum of squares).

Effect type	Variation source	Fluctuating asymmetry		Leaf length		Specific leaf area	
		Statistics	*p*	Statistics	*p*	Statistics	*p*
Fixed	Treatment (T)	*F*_1,54.4_ = 0.36	0.55	*F*_1,54.0_ = 0.88	0.35	*F*_1,54.0_ = 7.69	0.0076
	Species (S)	*F*_2,54.4_ = 0.30	0.74	*F*_2,54.0_ = 91.0	< 0.0001	*F*_2,54.0_ = 14.4	< 0.0001
	T × S	*F*_2,54.4_ = 0.67	0.51	*F*_2,54.0_ = 0.82	0.82	*F*_2,54.0_ = 0.14	0.87
Random	Individual	*χ* ^2^_1_ = 0.21	0.32	*χ* ^2^_1_ = 311.3	< 0.0001	—	—

### 3.3 Among‐Plant Variation

Individual plants within each of the three willow species differed significantly in leaf size but not in leaf FA (Table 2).

## 4 Discussion

Previous experiments conducted on *Vaccinium myrtillus* (Llorens et al. 2002) and *Betula utilis* (Xu et al. 2012) indicated that the application of a warming treatment (1°C for *V. myrtillus* and 2.9°C for *B. utilis*) to alleviate cold stress decreased FA. In contrast, in the present study, even though our applied warming treatment (1°C–2°C) was comparable, it did not decrease FA in any of the three studied willow species. Four methodological explanations could account for this ‘negative’ result. First, the temperature elevation in our OTCs may have been too small or it may have been mistimed relative to the period of greatest plant sensitivity. Second, willows may be inherently less sensitive to cold stress than birches—the genus that has provided most of the evidence for temperature‐driven FA changes (Valkama and Kozlov 2001; Xu et al. 2012; Zverev and Kozlov 2020; Shadrina et al. 2023). Third, a high measurement error and correspondingly low repeatability may have obscured subtle treatment effect and prevented its reliable detection. Fourth, the ‘positive’ results reported in the previous studies may have emerged due to confirmation bias.

The first two potential explanations are undermined by two of our findings. One is the significant increase in SLA among the OTC‐enclosed willows (Figure 2g–i). The other is the higher survival rates of leaf beetle (*Chrysomela lapponica*) larvae feeding on the OTC‐enclosed plants than on unenclosed controls (E. Zvereva, pers. comm.). Together, these observations confirm that the willows are sensitive to the OTC‐induced temperature elevation. However, the changes observed in willow leaves cannot be unambiguously interpreted as indicators of reduced cold stress, as SLA and herbivory responses are known to vary with stress type and plant species.

Reductions in SLA (i.e., the production of thicker, denser leaves) are often observed under a range of stress conditions, including drought, intense light or limited nutrients—conditions that select for more robust leaf structures (Wright et al. 2001; Poorter et al. 2009). In contrast, SLA tends to increase in response to elevated temperatures, shading or high resource availability—conditions that may either alleviate or intensify stress (Poorter and Bongers 2006; Griffin‐Nolan et al. 2018). Similarly, host plant stress can have either positive or negative effects on insect herbivores, depending on the ecological context (Price 1991; Koricheva et al. 1998). Nevertheless, despite this complexity, several lines of evidence suggest that growth in our OTCs likely alleviated, rather than intensified, environmental stress—particularly cold stress—in willows.

First, our study site lies just 1 km from alpine habitats and only 100–150 km south of the northern range limits of the studied willow species, whereas their southern limits extend 1500–3000 km farther south (www.iNaturalist.org). This biogeographical pattern implies that a warming of 1°C–2°C was likely to bring the local climate closer to the willows' optimum. Second, previous studies have shown that experimental warming either benefits *S. myrsinifolia* or has no adverse effects (Veteli et al. 2002; Sivadasan et al. 2015). Third, although not statistically significant, the observed increase in leaf size under the OTC treatment further suggests a release from a climatic limitation. Thus, although willows do respond to temperature elevation within OTCs, their responses do not include changes in FA.

We determined that the measurement error contributed 2%–10% to the total variation in leaf measurements used in the FA calculations. These values correspond to the uppermost quarter of the ME5 values summarised by Bechshøft et al. (2008) across FA‐related studies and are smaller than the average of 20% obtained in a study addressing the reproducibility of FA in 10 plant species (Kozlov et al. 2017). Therefore, we consider that measurement accuracy is an unlikely reason for the absence of the expected decrease in willow FA in our experiment.

Taken together, our results align with previous blinded studies that reported no consistent FA responses across environments and that attributed earlier ‘positive’ findings to confirmation bias (Májeková et al. 2024; Kozlov 2025). Our results also reinforce the conclusion that the response to environmental stress is generally lower for FA than for other morphological or physiological indicators (Black‐Samuelsson and Andersson 2003; Fair and Breshears 2005; Kolbas et al. 2014; Francis and Gilman 2019; Kozlov et al. 2022). These results support Kozlov's (2017) view that the perceived high sensitivity of FA is largely a myth—one rooted in isolated cases of increased FA occurring without observable declines in organismal performance (as reported by Llorens et al. 2002).

## 5 Conclusions

A careful evaluation of our data set—collected and processed in accordance with established protocols for both studies of FA (Palmer and Strobeck 2003; Graham 2021; Kozlov 2025) and field ecological research in general (Filazzola and Cahill 2021)—reveals no significant differences in leaf FA between willows grown inside OTCs and those exposed to ambient conditions. This finding, when viewed alongside other methodologically rigorous studies, reinforce previous evidence (Kozlov 2017, 2025; Májeková et al. 2024) that FA is not a reliable indicator of increased stress, and further demonstrate that it also fails to reflect stress release.

The considerable delay in publishing our results obtained 25 years ago is a typical example of time‐lag publication bias, resulting from a previously prevailing reluctance to report non‐significant findings (Franco et al. 2014; Showell et al. 2024), particularly those that challenged widely accepted concepts. We urge researchers studying FA in living organisms to publish well‐conducted studies with non‐significant, negative or inconclusive outcomes, as this will create a more balanced and accurate representation of the available evidence.

To strengthen our findings, we deposited images of all leaves included in this study (Data S1 in Data Availability section) in an open access repository. We invite all scientists investigating FA in living organisms to support this initiative of sharing images of study material, thereby enabling data quality control and ensuring repeatability.

## Author Contributions

**Dmitry E. Gavrikov:** investigation (equal), methodology (equal), writing – review and editing (equal). **Vitali Zverev:** investigation (equal), methodology (equal), writing – review and editing (equal). **Mikhail V. Kozlov:** conceptualization (equal), data curation (equal), formal analysis (equal), methodology (equal), visualization (equal), writing – original draft (equal).

## Funding

M.V.K. and V.Z. were supported by Maj ja Tor Nesslingin Säätiö and the Suomen Akatemia (project 362731).

## Conflicts of Interest

The authors declare no conflicts of interest.

## Data Availability

Images of all leaves used to assess FA and other traits (Data S1), along with the results of all measurements (Data S2 and S3), are available on Dryad: DOI: https://doi.org/10.5061/dryad.n8pk0p38b.
